# A Qualitative Study Identifying Barriers and Facilitators of Physical Activity in Rural Communities

**DOI:** 10.1155/2019/7298692

**Published:** 2019-06-23

**Authors:** Amanda S. Gilbert, Dixie D. Duncan, Alan M. Beck, Amy A. Eyler, Ross C. Brownson

**Affiliations:** ^1^Prevention Research Center in St. Louis, Brown School at Washington University in St. Louis, St. Louis, MO, USA; ^2^Department of Surgery (Division of Public Health Sciences) and Alvin J. Siteman Cancer Center, Washington University School of Medicine, St. Louis, MO, USA

## Abstract

**Background:**

Physical activity (PA) rates are lower in rural populations, compared to urban and suburban counterparts. Since PA is shown to decrease the risk of cancers and chronic diseases, increasing PA in rural environments is an important disease-prevention strategy. However, in order to develop effective interventions for rural populations, more research is needed. The purpose of the study was to elicit rural residents' thoughts and perceptions related to PA and walking trail use.

**Methods:**

Key informant interviews were conducted via telephone, with 62 adults, living in six rural communities in southeast Missouri, who identified as stakeholders, walking trail users, and nontrail users. Participants were recruited through word of mouth and snowball sampling. Interviews were digitally audio-recorded, transcribed, and qualitatively analyzed.

**Findings:**

Analysis revealed variation within the rural population, with each town unique in what constituted barriers and facilitators to PA. Life priorities other than physical health were found to be important motivators to PA and also influenced how PA was obtained. Community size was found to impact community resources and infrastructure, although this was mitigated by poverty rates.

**Conclusion:**

Rural communities are distinct from one another with different views and approaches to PA. Future interventions designed to increase PA should be mindful of differences at the individual and town levels and avoid a one-size-fits-all approach. Interventions would benefit from insight and support from community members and stakeholders, to facilitate a tailored approach to increase PA.

## 1. Introduction

In February 2018, the Physical Activity Guidelines Advisory Committee submitted its scientific report to the Department of Health and Human Services, providing further evidence for the positive relationship between physical activity (PA) and health outcomes [1]. The report builds on previous research indicating an increase in PA reducing the risk of chronic diseases, certain cancers, and all-cause mortality [2–8]. Strong evidence exists that an increase in PA is associated with a risk reduction of 10 to 20 percent for developing multiple forms of cancer including bladder, breast, endometrial, adenocarcinoma of the esophagus, gastric, renal, lung, and colon [1]. There is a reduced risk of developing other noncommunicable diseases, such as type II diabetes, coronary artery disease, high blood pressure, stroke, and metabolic syndrome with increases in PA [2]. Since many of these health outcomes are leading causes of death in the United States (US) (e.g., cardiovascular disease and cancer), PA is an important priority for research.

In spite of the known health outcomes, a large proportion of adults do not get the recommended amount of PA, and these rates vary by population subgroup and living environment. Some US communities, such as those in rural areas, have even lower rates of PA [9–11]. The prevalence of rural physical inactivity has been estimated at 24.1 percent, which is 50 percent higher than in urban communities [12, 13]. The vast difference in PA rates is significant, given rural populations make up 15 percent of the US population and have worse health outcomes than their urban counterparts [14]. Overall, individuals living in rural areas experience higher rates of colon and lung cancers, cardiovascular disease, type II diabetes, and obesity [12, 15–18]. The disparity between rural and urban populations is getting worse as the gap in life expectancy continues to widen [12, 19].

Despite the low rates of PA in rural communities, the majority of qualitative and quantitative research around increasing PA has been conducted in urban and suburban settings [20, 21]. The qualitative analyses conducted in rural populations have so far focused on definitions and language around PA, particular rural populations based on age, gender or location, and translation of validated PA measurement tools to the rural population [22–25]. Much of this research lags far behind the base of evidence for urban areas and is often limited by the variability in rural populations. As such, it is essential to understand PA within rural communities, especially given the continual growth in evidence demonstrating the positive impact of PA on health, in addition to the low rates of PA, and worse health outcomes in rural communities. Through community engagement by consulting rural residents and stakeholders, this study aims to address this need in two ways: first, by eliciting rural residents' perceptions about their experience with PA at local walking trails and in their communities, specifically around barriers and facilitators; second, by assessing community resources for the promotion of PA through stakeholder engagement.

## 2. Methods

### 2.1. Communities

Key informant interviews were conducted with residents of six rural communities in southeast Missouri. The communities were chosen as part of a larger intervention study, based on location, population size, and presence of at least one local walking trail. Walking trail used to promote PA is part of this larger study intervention and therefore a focus of this current study [26]. Rurality was defined by the US classification system whereby each community was at the nonmicropolitan level (less than 9,999) or micropolitan level (10,000 to 49,999) [27]. Communities chosen had populations ranging from 2,000 to 17,000. The Institutional Review Board at Washington University in St. Louis approved all study methodologies.

### 2.2. Key Informants

A purposive sample of adult residents, who were able to be physically active and lived within the six communities, were recruited to participate in the interviews. In order to understand PA in rural communities from multiple perspectives, informants came from one of three groups, stakeholders, walking trail users, and walking trail nonusers. Stakeholders were defined as influential residents working in positions within local agencies (e.g., local health departments, parks and recreation departments, and city councils). Stakeholders were recruited through word of mouth sampling. In order to explore experiences and perceptions regarding trail use, both local trail users and residents who did not use the local trails were included. Trail users self-identified as residents who reported regular use of available trails. Trail users were recruited at the trails, through word of mouth and snowball sampling. Nontrail users were defined as residents who lived within 2 km of available trails and reported not using these trails. Nontrail users were recruited through word of mouth, snowball sampling, and fliers.

### 2.3. Interview Guide

Interview questions were used to elicit information about PA in rural communities and were informed by scientific reviews and previous experience in PA [28–33]. Questions were tailored for each of the three groups of informants. The first part of the interview included background information, current PA behavior, and perceptions and social norms of PA within their community. The second part of the interview assessed perceived barriers and facilitators of PA. Trail users were asked more specifically about reasons they used available trails, while nontrail users were asked about reasons they did not use available trails. Open-ended and direct questions were used and included examples such as

  “What keeps you from using the trail?”
  “Does weather keep you from using the trail?”
  “Can you think of anything that would encourage you to use a walking trail?”
  “What do you like most about the trail?”
  “Do you normally walk alone or with others at the trail?”

Questions were also asked about PA promotion, community events, and events specifically geared towards PA in their communities. These questions were meant to gain insight into community support and available resources for PA. Stakeholders were asked additional questions about their organizations background, available resources for PA, and role in promoting PA in the community.

Interviews with residents were conducted over the telephone and lasted 30–60 minutes. Residents were interviewed until saturation was reached and no new information was being obtained. A $20 gift card was provided as an incentive upon completion of the interview. Interviews were digitally audio-recorded and transcribed.

### 2.4. Analysis

Transcripts of the interviews were qualitatively analyzed with NVivo [34]. Two codebooks were created, one for stakeholder interviews and another for trail user and nontrail user interviews. The purpose of the separate codebook for stakeholders was to capture additional information provided by stakeholders about organizations and available resources. The codebooks were organized to explore major themes and topics presented in the interviews. Six interview transcripts, three stakeholder interviews, and three trail user and nontrail user interviews were first read over by two members of the research team and analyzed using initial codebooks. Analyses were compared by both team members, and an evaluation of discrepancies was also conducted by a third team member. After careful consideration and discussion among the three team members, adjustments were made to each codebook to address any ambiguity in the codebooks and process of analysis. All transcripts were coded and analyzed with the updated codebooks. The team members then discussed each coded transcript in detail to ensure dependability and accuracy in coding.

## 3. Results

### 3.1. Demographics

In total, 62 residents were interviewed, consisting of 32 stakeholders, 13 nontrail users, and 17 trail users. Demographics were obtained for race and gender. Overall, 79 percent (*n*=41) of participants were identified as Caucasian, 21 percent (*n*=13) as African American, and 71 percent (*n*=44) as female (Table 1).

### 3.2. Community Resources: Infrastructure

Community resources and structure varied depending on the community size. Stakeholders from smaller towns (i.e., 6,000 or less) identified fewer available resources in the community and a reliance on informal organizations to promote PA. Stakeholders from larger towns (i.e., 10,000 or more) identified greater available resources and more formal organizations to promote PA. Availability of resources and more formal infrastructure were less present in larger communities with a higher rate of poverty. All stakeholders reported walking for PA is perceived as normal in their community, and some stakeholders noted PA behaviors have become more accepted in the past 5–10 years.

### 3.3. Barriers to PA

The most commonly reported barriers by stakeholders in all communities were individual-level barriers. These included lack of motivation to exercise, lack of understanding regarding impact of PA on health, and lack of knowledge on how to exercise properly. While stakeholders cited internal barriers as the most common barriers to PA, trail users and nontrail users tended to find environmental barriers to be a greater obstacle to PA. These environmental barriers to PA included lack of desired trail amenities and characteristics, weather, location and accessibility, and safety. Individual barriers, cited less often by trail users and nontrail users, included lack of motivation, time, and finances. Trail users and nontrail users did not differ much on identification of barriers to PA (Tables 2 and 3).

The identification of environmental barriers to PA differed by community. Safety was cited as a barrier to PA in only two of the communities, and location and accessibility were identified by only one community. Respondents in all but one community indicated trail amenities and characteristics as a barrier to PA.

### 3.4. Facilitators to PA

Trail users and nontrail users indicated different facilitators to PA. Trail users identified social and mental well-being as primary sources of motivation for PA, while nontrail users cited physical health and weight loss. This corresponded with trail users reporting participating in PA more often with others, while nontrail users reported a tendency to participate in PA alone. Trail users also mentioned safety and trail amenities as facilitators to PA, which were not indicated by nontrail users. Trail users and nontrail users equally indicated individual and environmental facilitators to PA (Tables 2 and 3).

Facilitators to PA differed among the communities. Safety was a facilitator in only one community. This community was also the only one in which the top three facilitators indicated were environmental. There were three communities in which physical health was reported as a facilitator. Social well-being was identified as a facilitator in all towns, with mental well-being indicated in all communities except one.

### 3.5. Ideas for Increasing PA at the Community Level

When asked what events would promote PA and encourage community members to attend events at trails in their community, stakeholders most commonly reported competitive running events, family focused events, park clean up days, resource and wellness fairs, social cause events, and free food events. Trail users and nontrail users were asked what would most encourage others to get PA. Both trail users and nontrail users identified education about PA to be the most effective way of increasing PA in others. Trail users also noted promotion of mental well-being and enjoyment of nature to encourage PA in others, differing from nontrail users identification of convenience and available indoor places for PA (Table 4).

## 4. Discussion

The purpose of this study was to gain insight into PA and trail use in rural communities from the perspective of local residents. The aim was to understand PA behaviors, barriers, facilitators, and community resources to inform future interventions for increasing PA in rural communities. The information obtained through the interviews highlights the diversity between rural communities around PA, specifically, what constitutes barriers and facilitators to PA. The barriers and facilitators identified in one community were not necessarily the same barriers and facilitators identified in another. Barriers such as safety, location, and accessibility were not uniformly present throughout all rural communities. Similarly, facilitators such as trail amenities and characteristics and physical health were not present or prominent in most communities. Understanding that not all rural communities are the same regarding PA is important for future PA interventions in rural settings. Community engagement is therefore necessary to better understand how PA is experienced to honor and respect the unique experience of each rural community [35]. The engagement will allow for a tailored PA intervention as well as a higher probability of success in increasing PA.

When asked about ideas for events to promote PA and what would encourage others to be physically active, a consistent theme was life priorities outside of health. These motives were identified via ideas for community events to promote PA, what respondents thought would encourage others to get PA and what facilitated PA among the respondents. Suggested community events focused on family, social activities, and events with a social cause, aligning with previous research findings of social support and the social environment to be a strong influence on PA [36–38]. Ideas of encouraging others to get PA focused on enjoyment of nature and mental well-being. Facilitators to PA were broad and not solely focused on physical health, with mental well-being and social well-being often cited by respondents. These findings support recent research in which positive feelings provided greater motivation for PA than good physical health [39]. Utilizing life priorities and other facilitators, aside from physical health, as motivators for PA is important in reaching the large group of individuals who are less likely to get PA. The focus on PA motives outside of physical health builds on recent research in which PA is viewed through the lens of a complex causal framework [40]. The determinants of increased PA are varied and multifaceted, supporting the notion that PA can be experienced and thus increased in many different ways. As such, future PA interventions would benefit from promoting PA through these life priorities. An approach solely focused on physical health may not be of benefit to individuals who may relate to PA differently and be motivated for nonhealth reasons.

The ways in which residents engage in PA were impacted by their motives for PA. Respondents who more often identified social and mental well-being as facilitators to PA were also more likely to be trail users than nontrail users. Conversely, respondents who more often cited physical health and weight loss were more likely to be nontrail users. These findings are important, given Thomas Park et al. found people in this geographic region were more likely to meet recommended PA guidelines if they were trail users [41]. Understanding nontrail user's motivations of physical health can inform interventions aiming to promote trail use, in this case, by focusing on the physical health benefits of being outdoors and using trails.

Finally, the study found community size influences available resources and infrastructure. Taking resources and infrastructure into account can be an important aspect to consider when engaging a community and determining how to best implement a PA intervention. Smaller rural communities (i.e., less than 6,000 residents) had fewer resources and less infrastructure and may require different types of interventions than larger rural communities. As noted previously, larger communities tended to have a strong infrastructure and more readily available resources. However, it is important to note poverty was found to mitigate the effect of size. Larger communities with higher poverty rates were more similar to smaller communities in regard to available resources to promote PA and infrastructure. These findings support recommendations for prioritizing funding in low-resource communities and creating coalitions for capacity building.

## 5. Limitations

There are several limitations inherent to exploratory qualitative studies. First, due to the variability in rural communities emphasized in this study, findings may not be transferable to other rural areas. Further, demographic data such as age and socioeconomic status were not collected, limiting analysis of these factors. Finally, social desirability bias may be present around PA behavior and affect accuracy of interview responses. Aside from these limitations, the study possesses important strengths. The aims of the study are appropriately addressed through a qualitative approach, and the study provides a full and meaningful assessment of PA by including perspectives of stakeholders, trail users, and nontrail users. In spite of these limitations, the results add to a sparse body of the literature on rural PA and can help future studies for planning interventions in these areas.

## 6. Conclusion

This study provides important information about how PA is perceived in rural communities and the ways in which these perceptions might be used to design and improve PA interventions in rural settings. The findings support the idea that rural communities are not homogenous in how PA is experienced. On both individual and community levels, facilitators and barriers to PA are experienced in different ways. Future interventions would benefit from tailored approaches employing community engagement. Going beyond consultation and promoting collaboration would allow for a better understanding and more effective approach to barriers and facilitators to PA specific to that community. Further, interventions addressing the various ways PA is experienced on an individual level, from social and mental motivations to physical health, will have a broader reach. Life priorities such as spending time with family and friends and enjoying nature are useful avenues for prompting PA and support the varied ways in which PA is experienced. The findings in this study support the previous research; however, more research is needed to explore how these varying ways of experiencing PA can best be used to promote PA.

## Figures and Tables

**Table 1:** Demographics.

	Stakeholders, *n*=32	Trail users, *n*=17	Nontrail users, *n*=13	Total, *n*=62
Race				
Caucasian	93.75% (30)	64.71% (11)	61.54% (8)	79% (41)
African American	6.25% (2)	35.29% (6)	38.46% (5)	21% (13)

Sex				
Female	68.75% (22)	88.24% (15)	53.85% (7)	71% (44)
Male	31.25% (10)	11.76% (2)	46.15% (6)	29% (18)

**Table 2:** Trail user and nontrail user themes.

Theme	Subthemes	Definition	Examples
Attitudes and perceptions of PA	(i) Where and how do other people get PA? (ii) What are the barriers and facilitators for other people to get PA?	The participants' perceptions of how other people in the community experience PA.	“I think some people are just lazy” “I notice a lot of people walking.” “Maybe if there was more information put out, flyers, or something, that people could see. Then we would know more by knowing where the trails are.”

Barriers to PA	(i) Environmental (a) Location and accessibility (b) Safety (c) Trail amenities/characteristics (d) Weather (ii) Individual (a) Financial (b) Psychological (c) Time	(i) Environmental Physical characteristics of the trail such as shade, lighting, benches, exercise stations, accessibility and safety. (ii) Individual concrete factors such as high gym fees and available time for PA. Psychological factors such as a lack of interest, knowledge or motivation, and feelings about what PA means.	“Shelby trail has zero shade. I mean zero.” “If I get busy or something, or working late, or the kids might have me busy. That's the only thing. Yeah, that's the only problem.” “It's been hot this summer, just trying to find a time to get out there where it's not scorching hot.”

Facilitators to PA	(i) Environmental (a) Location and Accessibility (b) Safety (c) Trail Amenities/Characteristics (ii) Individual (a) Mental well-being (b) Social well-being (c) Physical health (d) Weight loss	(i) Environmental helpful trail amenities such as benches, water fountains, and exercise stations. Easily accessible, safe from crime. (ii) Individual factors such as enjoying walking with others, socializing while getting PA, feeling relaxed, and enjoying being outside.	“It's got benches if you get tired or if you get winded you can sit down.” “Well, again, the close proximity to my home. I'm only a hop, step, and a jump from there, so it's easy, plus the parking is very easy.” It relieves a lot of stress for your mind. It gives you time to think about stuff and it's something to do. I do it a lot because it clears my mind.”

**Table 3:** Physical activity comparison of trail users and nontrail users.

	Trail user	Nontrail user
Individual PA		
Alone	−	**+**
With others	**+**	−
Motivated by social and mental well-being	**+**	−
Motivated by physical health	−	**+**

Effective PA promotion		
Education	**+**	**+**
Available indoor places	−	**+**
Outdoor places	**+**	−

+: reported. −: not reported.

**Table 4:** Stakeholder themes.

Themes	Subthemes	Definition	Examples
Organization	(i) Participant role (ii) Participant background (iii) Organization background	Stakeholder's role in organization and how that organization operates and contributes to PA in the community. Also includes organization's place in larger community infrastructure.	“I'm the City administrator here…have been so for the last eight and a half years, a total of 13 years working in state government.” “The county health (department) is responsible for promoting and protecting the health of its citizens.”

Existing PA events and promotion	(i) PA event and description (ii) No PA events (iii) Other organizations promoting PA (iv) Community events	Community events or promotion that contains an element of PA. Other organizations that already promote PA.	“Related to physical activity we have the 5^th^ annual Kiwanis Wolf Creek Trail Race.” “We have sports programming for kids and adults, all the various kinds of team sports. We also promote our local trails and greenway.”

Ideas for future events, partnerships and Promotion		Stakeholder's knowledge around potential ways to promote PA and possible collaborations within the community.	“In this area if you offer food and free fun then you're pretty bound to get some folks there. As long as it's free. As long as it's publicized in the right areas and offer food.”

Community information		Community information related to culture, communication, logistical patterns, governance, and infrastructure.	“It's a community of 2,000 people so we're pretty much connected in all kinds of ways and settings. We always have something that we are either responsible for, participating in, or planning for.”

## Data Availability

The transcripts used to support the findings of this study have not been made available due to confidentiality.
